# Improved processing workflow and student transparency with student mistreatment reports leads to graduation questionnaire data gains

**DOI:** 10.12688/mep.20444.1

**Published:** 2024-08-12

**Authors:** Adam Channell

**Affiliations:** 1 Homer Stryker MD School of Medicine, Western Michigan University, Kalamazoo, Michigan, 49008, USA

**Keywords:** Student mistreatment, Undergraduate medical education

## Abstract

Mistreatment of students has been historically documented as common in U.S. medical schools, but graduate questionnaire (GQ) data from the Association of American Medical Colleges (AAMC) displays high numbers of students who have experienced mistreatment but not reported the incident. There are many reasons within the literature as to why students do not report their experiences, including fear of academic repercussion or a misunderstanding of what constitutes as mistreatment. Our institution found through GQ data that there was a shortcoming in understanding policies and knowledge of procedures associated with mistreatment, and student focus group responses showed that many students were not confident that their reports would receive follow-up on the part of the institution. These factors led to the formation of a task force to investigate our school’s workflow once a report of concern for mistreatment is received and examine measures to increase transparency to the student body that their reports are acted upon. We took measures to place a greater emphasis on communication with students during the mistreatment report workflow, as well as releasing name-blinded data within our weekly student communication emails regarding reports that had been processed and resolved. The results after one year of these efforts saw our GQ percentile data jump from falling between the 10 ^th^ to 25 ^th^ percentile to the 90 ^th^ percentile for student awareness of mistreatment policies and from between the 25 ^th^ to 50 ^th^ percentile to between the 75 ^th^ to 90 ^th^ percentile for student knowledge of mistreatment procedures. These jumps in GQ figures provide insight for policy changes that could benefit other institutions struggling with building a safe environment for students to confidently report incidents of mistreatment with knowledge that their concerns are important and acted upon.

## Introduction

Student mistreatment in undergraduate medical education is a well-documented issue ^1^ that can affect students’ physical and emotional well-being and hinder confidence in learning and clinical abilities ^2– 4^. Since 1978, the Association of American Medical Colleges (AAMC) conducts a yearly graduate questionnaire (GQ) survey which provides individual schools data on their performance against national composite score. The de-identified data from the GQ is available on the AAMC public website and does not require a copyright license. Beginning in 1991, questions regarding student mistreatment were added that allowed researchers to investigate issues that were formerly ignored as part of the norm while in medical school ^5^. The literature contains figures that range between 16–50% of students reporting experiencing mistreatment of some sort during their four years of medical school ^4, 6, 7^. However, national AAMC GQ data also shows that among students who claimed they had been mistreated, 80% said that did not report the incident ^6^, revealing a persisting issue of underreporting of cases of mistreatment ^8^. The national statistics trend aligned with our institution’s data found in the GQ survey and student focus groups.

The literature contains several examples of obstacles students face that lead to underreporting of mistreatment incidents. These include a lack of clarity from the school to early students as to what incidents qualify as mistreatment, feeling student claims may be dismissed, and fear of retribution or retaliation for reporting ^5, 9– 11^. A 2020 study reported that if students do not see concrete evidence that their mistreatment reports are being acknowledged and acted upon, they will be less likely to report mistreatment ^7^. As an interviewed student in their 2020 study notes, “I think the person who makes the report should have some sort of feedback about this is what was done. And it should be visible to the community, like to the medical students and the learners in general, that these reports do lead to change because right now it doesn't seem like there is”. We found similar feedback from responses in our student focus group interviews. Additionally, our GQ data showed that we had room for growth in terms of student awareness of our institution’s policies and procedures for student mistreatment.

## Methods

A task force was created at our institution to look at the existing mistreatment report processing flow. Prior to 2022, students were able to report mistreatment through an ad hoc form through our online portal, through end of course and clerkship evaluations, and through personal communication with faculty or other school officers via personal communication, email, or phone call. A faculty member in the department of medical education served as the funnel for any reports that were made, documenting data associated with the incident in a protected file. Finally, a name-blinded summary of the report was sent to course or clerkship leadership for potential feedback to any identified faculty members or for course improvement.

**Figure 1.**
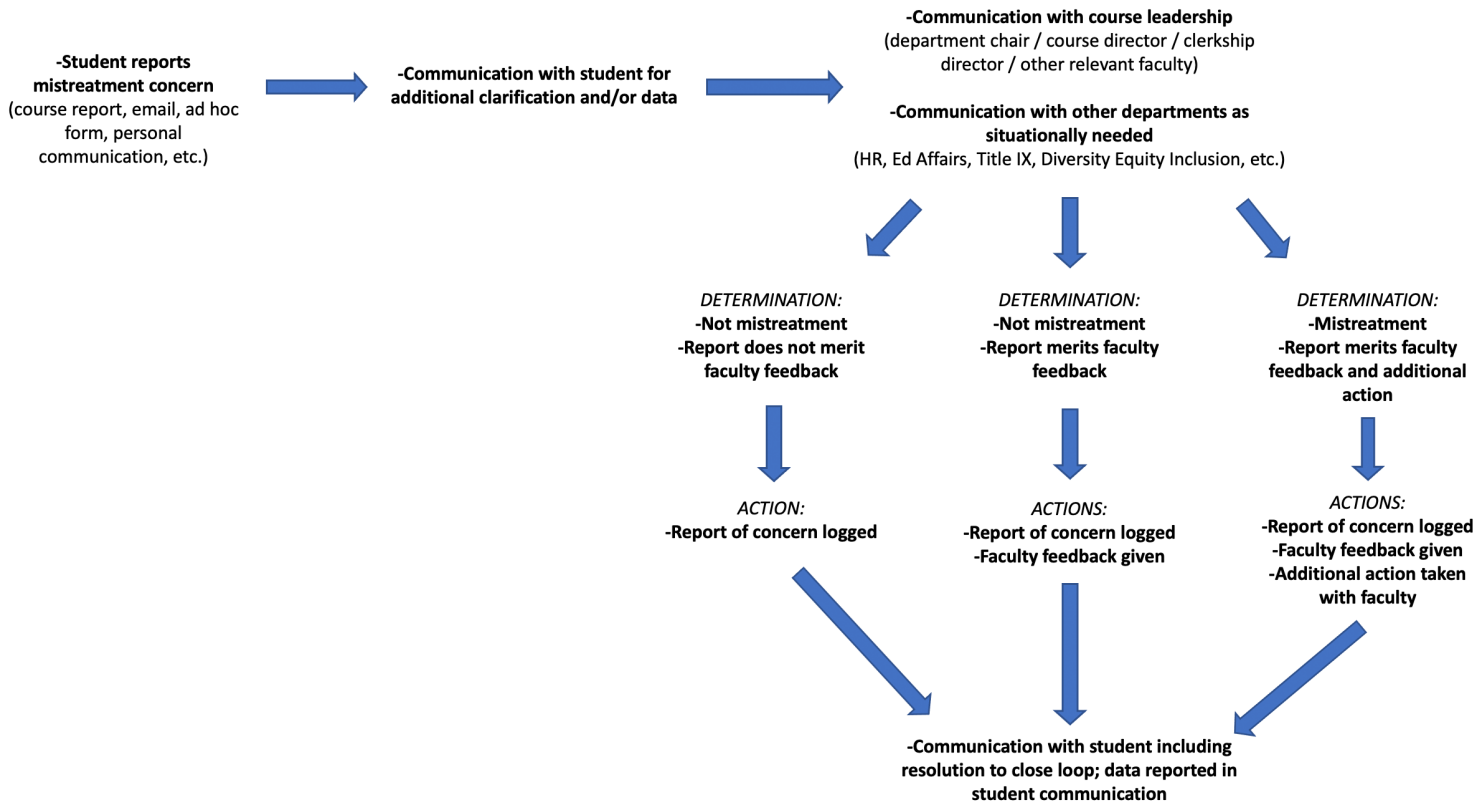
Institutional workflow upon receipt of student mistreatment report of concern.

**Figure 2.**
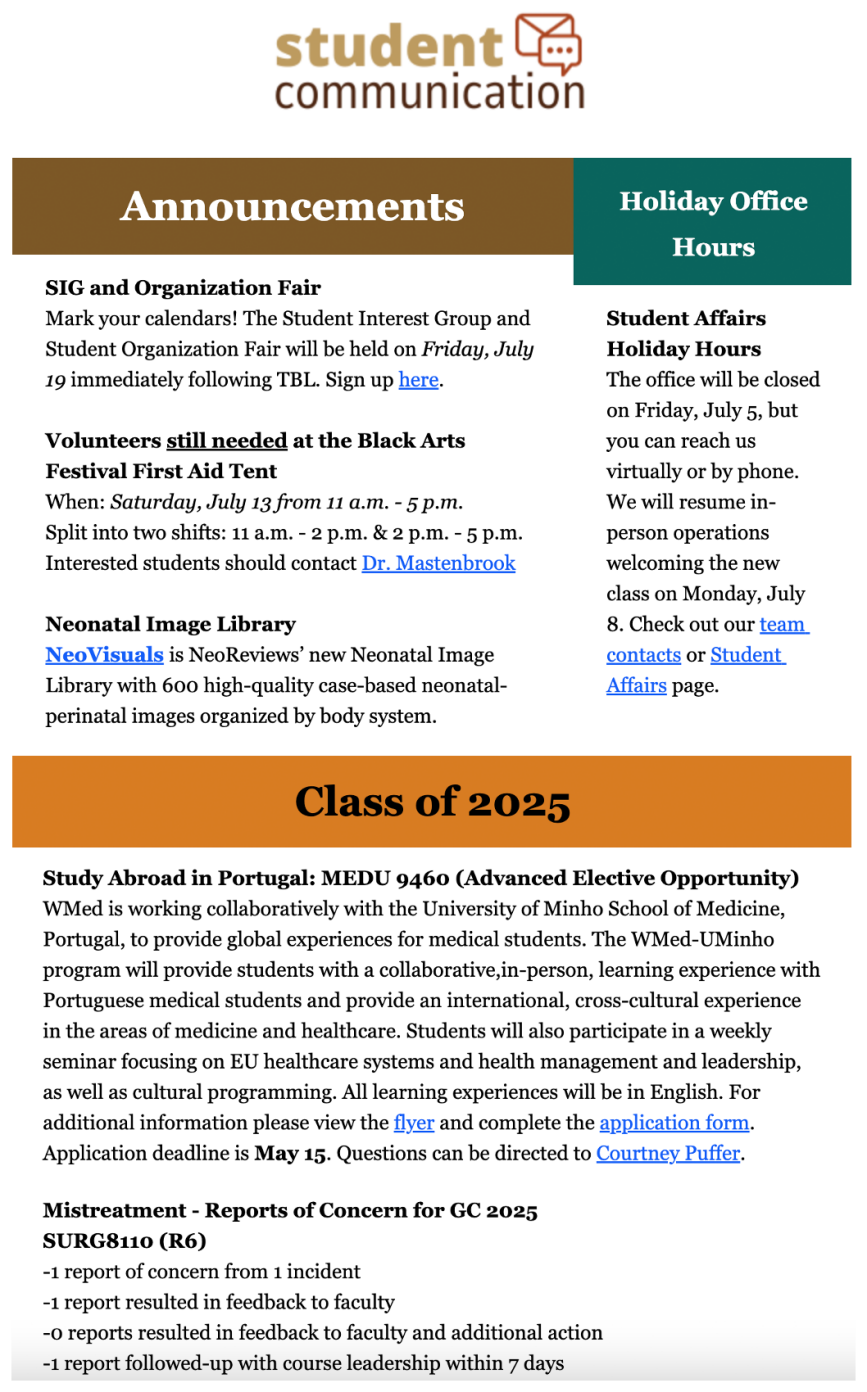
Example mistreatment report of concern posted within institution’s weekly student communication email.

Our task force found that the obvious piece missing in our workflow involved student communication and transparency. Often, students would be contacted for additional details if needed, but initial communication with students following the submission of a report was not the norm. This was improved by ensuring that every mistreatment report receives a follow-up email that the report had been acknowledged and was being handled in an anonymous fashion. Additionally, the student was made aware that they would be contacted regarding the resolution of the situation being reported (see Figure 1). While this looked to address transparency in our process with students who had submitted a report, the issue remained with students who were generally unwilling to submit reports due to belief that it would not be acknowledged. The task force consulted student council members from various graduating classes at the institution, and the decision was made to include anonymous summaries of all mistreatment reports within our weekly student communication newsletter (see Figure 2). The summary includes graduating class, course name or clerkship rotation, number of reports of concern and incidents, whether reports resulted in feedback to faculty or other additional action, and whether the reports were followed up with course leadership and the student reporting within 7 days.

## Results

Our GQ data from 2022 indicated that questions related to student understanding of policies and procedures for mistreatment of students, our institution had room to grow (see Table 1). Our institution placed between the 10 ^th^ and 25 ^th^ percentile for awareness of mistreatment policies and between the 25 ^th^ and 50 ^th^ percentile for knowledge of mistreatment procedures. Our task force took efforts to include student communication as the bookend components of our workflow once a mistreatment report of concern is submitted. Additionally, we began using the weekly student communication email to show anonymous mistreatment report data to the entire student and faculty bodies to demonstrate that reports are, in fact, acted upon and processed. After one year of these changes, we saw our GQ percentile data jump from being between the 10 ^th^ to 25 ^th^ percentile to the 90 ^th^ percentile for awareness of mistreatment policies and from between the 25 ^th^ to 50 ^th^ percentile to between the 75 ^th^ to 90 ^th^ percentile for knowledge of mistreatment procedures (see Table 2).

**Table 1.** 2022 National vs. Institutional GQ Data ^12^.

	10 ^th^ Percentile	25 ^th^ Percentile	50 ^th^ Percentile	75 ^th^ Percentile	90 ^th^ Percentile	Institution
**GQ Report item #37:** **Mistreatment Policies** Are you aware that your school has policies regarding the mistreatment of medical students? (Percent answering “Yes”)	94.4	96.7	98.4	99.3	100.0	95.6
**GQ Report item #38:** **Mistreatment Policies** Do you know the procedures at your school for reporting the mistreatment of medical students? (Percent answering “Yes”)	80.0	86.0	92.1	95.6	97.7	91.1

**Table 2.** 2023 National vs. Institutional GQ Data ^13^.

	10 ^th^ Percentile	25 ^th^ Percentile	50 ^th^ Percentile	75 ^th^ Percentile	90 ^th^ Percentile	Institution
**GQ Report item #37:** **Mistreatment Policies** Are you aware that your school has policies regarding the mistreatment of medical students? (Percent answering “Yes”)	94.4	96.7	98.4	99.3	100.0	100.0
**GQ Report item #38:** **Mistreatment Policies** Do you know the procedures at your school for reporting the mistreatment of medical students? (Percent answering “Yes”)	80.0	86.0	92.1	95.6	97.7	96.7

## Conclusions/Discussion

Our institution’s changes in mistreatment report workflow and transparency to students improved our percentile rank within GQ categories regarding student awareness of mistreatment policies and knowledge of mistreatment procedures. The jump in GQ percentile rankings demonstrate that our institution is moving toward providing students concrete evidence that their reports of concern are being acted upon, with hopes that this will continue to improve trust between students and institutional faculty ^7^. An area to monitor for future research is whether our efforts increase the number of reports of mistreatment, and whether this has to do with students feeling empowered to speak up. Conversely, another area to monitor is whether the transparency decreases the number of mistreatment reports due to faculty being more aware that reports associated with their courses are now in the open within the student communication.

We are also hoping to build upon this success by introducing a presentation to all incoming first-year students, where we are able to educate our new students about the workflow and transparency attempts, as well as better define the behaviors amongst our educational faculty that should be reported as mistreatment ^10^. The hope is that this presentation helps students build a base toward their initial state of sense-making of their new environment and behavior that might constitute mistreatment ^7^.

Our efforts are meant to improve student understanding of our institutional policies and procedures surrounding mistreatment, as well as the clarity that they exist in a safe space where their concerns are important and treated seriously in terms of follow-up. What these efforts do not address are the larger issues at hand with the historically steady rates of students encountering mistreatment during their four years of medical school ^1, 8^. Decreasing the amount of mistreatment in general is entirely more complicated effort for our institution to continually address and improve upon. The purpose of the steps we have taken thus far, and those we plan to make in the future, is to lower the number of students who are not reporting when they are mistreated ^6^ by making students feel empowered and willing to take action.

## Consent

Because the author is reporting on improvements in the workflow, the Institutional Review Board (IRB) has determined this scholarly activity to be non-human subjects research. Our institution does routinely practice process improvement, therefore we do inform students via policy there is an expectation their information will be used in a de-identified fashion for improvements to our medical education programs.

Since the author is utilizing de-identified information available on a public website, the Institutional Review Board (IRB) has determined this scholarly activity to be non-human subjects research. In reviewing the “frequently asked questions” on the AAMC GQ website, students are aware and given an opportunity to decline human subjects research participation ^14^.

## Data Availability

The data that support the findings of this study are openly available at: https://www.aamc.org/data-reports/students-residents/report/graduation-questionnaire-gq There are no supplementary data or materials associated with this article. The de-identified information from the AAMC GQ used in this article, which the IRB has determined to be non-human subjects research, is presented in Table 1 and Table 2. These data are openly available at: https://www.aamc.org/data-reports/students-residents/report/graduation-questionnaire-gq
