# Supplementary material for: Improved processing workflow and student transparency with student mistreatment reports leads to graduation questionnaire data gains
Source: MedEdPublish (2016). 2024 Sep 17;14:62. Originally published 2024 Aug 12. [Version 2] doi: 10.12688/mep.20444.2 (PMC12413609; doi:10.12688/mep.20444.2)
Supplement: Peer review history

# Reviewer report: Reviewer response for version 2

**DOI:** 10.21956/mep.22115.r42720

**Published:** 2025-09-15

**Authors:** Fiona Moir

**Affiliations:** 1 The University of Auckland, Auckland, Auckland, New Zealand

It was gratifying to read about the proactive approach taken by this institution to tackle a difficult, pervasive problem. This article is important as it highlights the importance of communication with students and ‘closing the loop’ about actions taken to engender belief in the reporting system - especially as reporting processes and follow up can commonly be unclear or invisible. As many institutions are currently exploring how to address these issues, I would recommend that the authors provide some additional information to enable replication of their excellent system. And that this work is indexed.   

 I would suggest referring to some more recent literature. For instance, the first 4 references cited, regarding the consequences of student mistreatment, are all over a decade old. Whereas the reference from 1998 shows that it is a longstanding problem, more recent references are also needed as evidence of the fact that it unfortunately continues to be an issue.

 The authors accurately outline some of the causes for under-reporting of mistreatment including fear of lack of confidentiality, uncertainty of reporting processes and no belief that action will be taken. Given that this is the main topic of this research, it might be worth unpacking obstacles to reporting in a bit more detail. For instance, ‘fear of retribution or retaliation’ could include fear of imminent academic consequences whilst being a student – but also fear of future career consequences and opportunities. There are also other factors which influence reporting such as the hidden curriculum and normalisation of toxic culture/behaviour.

 The results show an impressive improvement in student awareness of reporting policies and procedures. However, as with any similar study design, the authors cannot claim that this change is entirely due to their intervention – as there will always be confounders that cannot be taken into account (for instance, increased media coverage of this type of topic or other external environmental factors could influence reporting). Unless it was a cluster RCT. However, the authors could state that it is highly likely that their change in workflow processes contributed to this improvement. They may wish to include this point as a study limitation.   

 Regarding Figure 1, which clearly outlies the workflow - I would have appreciated more information about how much information a student receives. Is it just a standardised response that the concern has been logged (regardless of the determination and action taken)? Or is the student informed as to the determination made and the level of action taken? For instance, in one of the routes where it was determine at ‘not mistreatment’- does the student receive the information that this is how it was categorised, or are they just informed in the same way for all 3 categories  (“concern logged’’, “concern logged + faculty feedback given”, “concern logged + faculty feedback given + additional action taken”).

 In Figure 1, I would suggest that something about 'supporting the student' is added to the first action following on from a student reporting a concern. Whilst this undoubtedly occurs, my thoughts are that it would be important to also state it as part of the workflow text. We all know it is a dual process of investigating the complaint, whilst also providing the student with a range of resources/support - depending on their needs.  

 I appreciated the screenshot of the wording of the student communication in Figure 2. In terms of ‘SURG8110 (R6)’ – can you give more information about what this refers to please? If it is the placement where the concern was raised, how many students would have been placed there during the last week? As there is a limited pool of students who could have raised a concern in that particular placement in that particular time-frame, I imagine it would have to be a big enough student group so as not to unintentionally identify the student who reported. If there are concerns reported for several placements, are those clinical placements all outlined separately in this student communication? (e.g. SURG8110 2 concerns and actions taken -  and then another placement with its specific concerns and actions taken). Or are the placements (with the number of concerns and actions grouped together), so individual placements (and therefore student groups) are less identifiable?

 I would love the discussion to incorporate, even briefly, some of the more nuanced challenges with this topic. For instance, with anonymous reporting (where the person reporting can name another person, but withhold their own name) - the potential occurrence of false allegations and any alleged ‘perpetrators’ right of reply (‘natural justice’), or the legal and HR challenges of taking action with anonymous complaints. Or the issue of ‘reporting back’ to students, whilst maintaining student anonymity and accidental identification of complainants due to pinpointing the site of the reports etc..        

 Grammar: Top of page 12: “ *Decreasing the amount of mistreatment in general is entirely more complicated effort for our institution to continually address and improve upon*”.  The grammar in this sentence needs to be improved.      

 Thank you for this work and this manuscript. Inspirational to see such strong leadership and innovation.

Have any limitations of the research been acknowledged?

Partly

Is the study design appropriate and does the work have academic merit?

Yes

Is the work clearly and accurately presented and does it cite the current literature?

Yes

If applicable, is the statistical analysis and its interpretation appropriate?

Not applicable

Are all the source data underlying the results available to ensure full reproducibility?

Yes

Are the conclusions drawn adequately supported by the results?

Partly

Are sufficient details of methods and analysis provided to allow replication by others?

Partly

Reviewer Expertise:

Student wellbeing; bullying, harassment and discrimination; mental health'; clinician wellbeing; medical education

I confirm that I have read this submission and believe that I have an appropriate level of expertise to confirm that it is of an acceptable scientific standard.

# Reviewer report: Reviewer response for version 2

**DOI:** 10.21956/mep.22115.r43175

**Published:** 2025-09-05

**Authors:** Sunny Nakae

**Affiliations:** 1 California University of Science and Medicine School of Medicine, Colton, CA, USA

Solid report about a targeted intervention addressing a core issue around reporting mistreatment.  The sample workflow and newsletter examples are also helpful for current practitioners. Much of the existing literature or core beliefs within medical education is that students do not report due to fear.  This article affirms that students can be encouraged to report when transparency is practiced about responses to concerns.  This article contributes to practice by taking on the argument that schools cannot do more to encourage reporting.  This is a tangible strategy that was shown to make a difference at one institution.

Have any limitations of the research been acknowledged?

Yes

Is the study design appropriate and does the work have academic merit?

Yes

Is the work clearly and accurately presented and does it cite the current literature?

Yes

If applicable, is the statistical analysis and its interpretation appropriate?

Yes

Are all the source data underlying the results available to ensure full reproducibility?

Yes

Are the conclusions drawn adequately supported by the results?

Yes

Are sufficient details of methods and analysis provided to allow replication by others?

Yes

Reviewer Expertise:

Student affairs, pathways programs, enrollment management, student support, admissions, equity and inclusion.

I confirm that I have read this submission and believe that I have an appropriate level of expertise to confirm that it is of an acceptable scientific standard.

# Reviewer report: Reviewer response for version 1

**DOI:** 10.21956/mep.21877.r38387

**Published:** 2024-09-05

**Authors:** Emmanuel Essien

**Affiliations:** 1 University of Uyo, Uyo, ,AkwaIbom State, Nigeria

Generally, student feedback and reports are commendable measures encouraging management's proficiency in better administration and student-teacher relationships. However, the methods of approach and study design have a significant effect on the accuracy of these reports/feedback.

 This open access portal study design is really not proficient, because a student would likely not give honest negative reports on a teacher's maltreatment when their identities are revealed. 

 According to Chung *et al., *( 2018) 80% of the students did not participate in the exercise. This is likely attributed to the method of data collection. Anonymous sibmission of report will deal with sentiment of threats (fear) of the unknown.

 The online portal, does it expose the identity of the student? If so, then you are bound to ave false reports. In addition, students who did not particpiate in the exercise could be attributed to ineefective respose to previous exerciese; where reports have been sent on a particular maltreatment with promises to be tackled, yet they were unattended to.

 However, some students do not understand the importance of these exercise to them and better managerial positions. Therefore, it is pertinent that Intensive orientation be considered. In addition, please engage with one-one-one engagment with students on reports.

Have any limitations of the research been acknowledged?

Yes

Is the study design appropriate and does the work have academic merit?

No

Is the work clearly and accurately presented and does it cite the current literature?

Partly

If applicable, is the statistical analysis and its interpretation appropriate?

No

Are all the source data underlying the results available to ensure full reproducibility?

Partly

Are the conclusions drawn adequately supported by the results?

Partly

Are sufficient details of methods and analysis provided to allow replication by others?

Partly

Reviewer Expertise:

Fisheries and Hydrobiology, Statistics, Environmental Studies.

I confirm that I have read this submission and believe that I have an appropriate level of expertise to confirm that it is of an acceptable scientific standard, however I have significant reservations, as outlined above.

## References

[1] : Learning About Medical Student Mistreatment From Responses to the Medical School Graduation Questionnaire. *Academic Medicine* .2014;89(5) : 10.1097/ACM.0000000000000199 705-711 10.1097/ACM.0000000000000199 24667505

[2] Chung MP, Thang CK, Vermillion M, et al.: Exploring medical students’ barriers to reporting mistreatment during clerkships: a qualitative study. Med Educ Online. 2018; 23(1): 1478170. PubMed Abstract Publisher Full Text Free Full Text.10.1080/10872981.2018.1478170PMC599095629848223

# Response

**Published:** 2024-09-06

**Authors:** Adam Channell

**Affiliations:** Medical Education, Western Michigan University Homer Styker MD School of Medicine, Kalamazoo, MI, USA

I've put additional thought into this, and I'm wondering if my opening sentence gives the wrong impression to readers. I was attempting to show that there are lots of reasons students don't report mistreatment first with this set of statements:

 The literature contains several examples of obstacles students face that lead to underreporting of mistreatment incidents. These include a lack of clarity from the school to early students as to what incidents qualify as mistreatment, feeling student claims may be dismissed, and fear of retribution or retaliation for reporting ^5, 9– 11^.  However, these reasons were just to show that the literature has many examples. This may draw attention to the variety of issues regarding students not reporting mistreatment, when at our institution, the main reason I was attempting to draw attention to in the follow up sentence was that students did not understand our process and did not think their reports were acted upon:

 A 2020 study reported that if students do not see concrete evidence that their mistreatment reports are being acknowledged and acted upon, they will be less likely to report mistreatment7. As an interviewed student in their 2020 study notes, “I think the person who makes the report should have some sort of feedback about this is what was done. And it should be visible to the community, like to the medical students and the learners in general, that these reports do lead to change because right now it doesn’t seem like there is”. We found similar feedback from responses in our student focus group interviews. Additionally, our GQ data showed that we had room for growth in terms of student awareness of our institution’s policies and procedures for student mistreatment.

 My question for the reviewers is that maybe this issue can be solved by simply removing the opening statement about the other reasons students do not report mistreatment other than worry that their reports are not followed-up on? This may focus the statement to be clear that the issue we were attempting to address was student understanding of our processes and policies so that it was clear that their reports don't disappear into the ether. Any feedback would be appreciated!

# Reviewer report: Reviewer response for version 1

**DOI:** 10.21956/mep.21877.r38486

**Published:** 2024-08-30

**Authors:** Amanda Bell

**Affiliations:** 1 McMaster University, Ontario,, Canada

This is an interesting look at processes put in place at one institution to address shortcomings in awareness of policies and procedures around reporting mistreatment.  A key barrier identified through student focus groups was lack of confidence in follow-up on reports received.  A task force resulted in workflow that increased communication with students and provided regular reporting on mistreatment concerns to students.  These efforts saw improvement in AAMC Graduate Questionnaire responses regarding awareness of mistreatment policies and procedures.

 I applaud this institution for seeking, listening to and responding to student voices as they relate to mistreatment and concerns around reporting.  I did not notice a mechanism for anonymous reporting described at this institution, which is something learners often mention would facilitate reporting.  A challenge with anonymous reporting in this institution's workflow is that additional information cannot be sought from the reporter which may make classification of the incident as mistreatment or not more challenging.

 The introduction sets out the challenges of mistreatment and underreporting as well as barriers to reporting.  The institution showed self reflection on their own Graduate Questionnaire results.

 The methods clearly lay out the findings of the task force and the workflow that was created to manage mistreatment reports.  The inclusion of an example of the weekly student communication was helpful as an illustration of action to the reader and to students.  I do have some concerns that such reports may not fully protect the anonymity of the students, particularly in smaller rotations or at smaller clinical teaching sites.  It would be helpful to know if there is a time lag between when reports are made and when they are communicated in the weekly emails to students.  Are there any other steps that are taken to protect student identity (e.g. only reporting quarterly or grouping together all reports from all clerkship sites)?

 In your discussion section it is a stretch to conclude that the "jump in GQ percentile ranking demonstrate that our institution is moving toward providing students concrete evidence that their reports...are being acted upon".   The results certainly demonstrate a large increase in the awareness of policies and procedures for reporting mistreatment and that is to be commended.  The authors should consider tempering their statement with an inference that this is due to the increase in student trust in the institution to act on reports but the data they are using does not directly demonstrate their conclusion.  This may be a place to expand upon limitations of available data points and plans to collect more specific data to inform future directions.

 You may need to consider a survey more tailored to the experiences of your learners as it relates to reporting mistreatment.  The AAMC Graduate Questionnaire questions are limited to awareness of mistreatment policies and procedures.  This does not equate to willingness by students to report nor feeling safe and confident in the reporting process.  I would argue this is the ultimate goal of our institutions, to ensure learners feel safe in coming forward with concerns and trust the institution will respond.  This may result in an increase in reports, which is not necessarily a negative finding, because they are no longer staying quiet about their experiences.

 The presentation that you are planning to give to all incoming students will be helpful in furthering your goals and familiarizing learners with the reporting system.  There may be value in repeating or adding on to such a presentation later in the curriculum, closer to the start of clinical clerkships, given that mistreatment appears to be more prevalent in the clinical setting than the pre-clerkship (Abate and Greenberg 2023).

 This article could be enriched by a brief description of what follow-up generally entails with faculty members or department leads.  Sharing this information, at a general level, with students might also help strengthen their understanding of the processes and improve their trust in the institution if they understand what you have in place and what actions result from your efforts.

 In your final paragraph I would suggest changing the wording in the final sentence to the positive, rather than a double negative, to increase its impact, "the purpose...is to INCREASE the number of students who ARE reporting when they are mistreated..."

 Overall an interesting read and helpful contribution to the literature leaving room for exciting future exploration and improvement.

Have any limitations of the research been acknowledged?

Yes

Is the study design appropriate and does the work have academic merit?

Yes

Is the work clearly and accurately presented and does it cite the current literature?

Yes

If applicable, is the statistical analysis and its interpretation appropriate?

Not applicable

Are all the source data underlying the results available to ensure full reproducibility?

Yes

Are the conclusions drawn adequately supported by the results?

Partly

Are sufficient details of methods and analysis provided to allow replication by others?

Yes

Reviewer Expertise:

Distributed Medical Education, Learner Mistreatment

I confirm that I have read this submission and believe that I have an appropriate level of expertise to confirm that it is of an acceptable scientific standard.

## References

[1] "Incivility in medical education: a scoping review."Abate, Laura E., and Larrie Greenberg. *BMC medical education 23, no. 1 (2023): 24.* .10.1186/s12909-022-03988-2PMC983805536635675
